# Machine Learning Models for Predicting Key Performance Characteristics of High-Temperature THz Quantum Cascade Lasers

**DOI:** 10.3390/nano16110651

**Published:** 2026-05-22

**Authors:** Mihailo Stojković, Novak Stanojević, Aleksandar Milićević, Nikola Vuković, Dušan Topalović, Milan Ignjatović, Aleksandar Demić, Dragan Indjin, Jelena Radovanović

**Affiliations:** 1 School of Electrical Engineering, University of Belgrade, Bulevar kralja Aleksandra 73, 11120 Belgrade, Serbia; mihailostojkovicpkr@gmail.com (M.S.); ignjatovic@etf.bg.ac.rs (M.I.); radovanovic@etf.bg.ac.rs (J.R.); 2 Vinča Institute of Nuclear Sciences, National Institute of The Republic of Serbia, University of Belgrade, Mike Petrovića Alasa 12-14, 11351 Vinča, PO Box 522, 11001 Belgrade, Serbia; amilicevic@vin.bg.ac.rs (A.M.); dusan.topalovic@vin.bg.ac.rs (D.T.); 3 Center for Light-Based Research and Technologies COHERENCE, Mike Petrovića Alasa 12-14, 11001 Belgrade, Serbia; 4 School of Electronic and Electrical Engineering, University of Leeds, Woodhouse Lane, Leeds LS2 9JT, UK; a.demic@leeds.ac.uk (A.D.); d.indjin@leeds.ac.uk (D.I.)

**Keywords:** terahertz quantum cascade laser, machine learning, Artificial Neural Networks

## Abstract

In this work, we applied Random Forest (RF), Extreme Gradient Boosting (XGBoost), and Artificial Neural Networks (ANN) to predict key performance characteristics of quantum cascade lasers (QCLs), including material gain, current density, and emission frequency. By developing a machine learning-based surrogate modeling framework that replaces computationally expensive simulations of QCLs, we enable orders-of-magnitude-faster evaluation and optimization of a high-dimensional configuration space. The training dataset was generated using a numerical simulator based on the density-matrix transport model. By combining physics simulations with machine learning, we achieved reliable predictions of device characteristics, with standardized RMSE values ranging from 0.21 to 0.55 for RF, 0.16 to 0.51 for XGBoost, and 0.04 to 0.22 for the ANN model, demonstrating the superior predictive performance of the ANN across all investigated performance characteristics. The ANN was subsequently used to analyze the full configuration space defined by possible layer thicknesses and electric fields. Approximately 44 million configurations were evaluated in about five minutes, achieving a speedup of approximately 90,000 times over the numerical simulator for a single configuration. This approach allowed the identification of designs with improved material gain and facilitated the efficient optimization of key parameters while maintaining high prediction reliability.

## 1. Introduction

Terahertz quantum cascade lasers (THz QCLs) [1,2] emit radiation in the far-infrared spectral region, a range that has long been difficult to access. This capability has boosted interest in their applications, such as medical diagnostics, chemical sensing, and imaging technologies [3,4,5,6,7,8,9,10]. To date, THz QCL emission has been demonstrated over a broad frequency range, from 1.2 THz [11,12] up to 6.03 THz [13] in pulsed mode, and from 1.2 THz [11,12] up to 5.71 THz [13] in continuous-wave (CW) mode. Additionally, device operation has been reported at temperatures as high as 261 K under pulsed conditions [14], while CW operation has reached 129 K [15]. In terms of performance, maximum output powers of 312 mW in CW mode [16] and 2.4 W in pulsed mode [17] have been achieved.

One of the most common active region designs is the Longitudinal Optical (LO) phonon-based design [18], known for its superior high-temperature performance. THz QCLs operated below 200 K in pulsed mode [19], until a new record of 210 K was achieved [20] using a high-barrier (x = 0.25) two-well LO-phonon design. Recently, alternative design concepts that shift the operation of LO-phonon structures by utilizing radiative transitions further from the GaAs LO-phonon resonance energy of 36 meV have emerged [21,22]. This approach has enabled significantly better high-temperature performance, with operation reported above 250 K [14,23].

This work centers on the recently demonstrated high-temperature LO phonon THz QCL that achieved pulsed operation at 261 K [14]. Its active region contains only two quantum wells, with barriers featuring an increased Al mole fraction (x = 0.35) compared to standard THz QCLs. The LO-phonon extraction energy is set at 48.5 meV, which is significantly higher than the 36 meV LO-phonon resonance. Electron–LO-phonon scattering is a key factor at high temperatures, particularly in the 10–70 meV range, peaking at 36 meV in GaAs [22]. The primary limitation for high-temperature operation is the non-radiative nature of LO-phonon scattering, which diminishes the population inversion between lasing states (typically 8–20 meV). Moreover, it has previously been reported that THz QCLs are sensitive to background doping, interdiffusion, and layer-thickness fluctuations, which can significantly affect the robustness of high-temperature QCL designs [24,25].

The design of new QCL structures is crucial for improving performance and expanding operational limits, which drives the development of advanced computational optimization methods. Various computational techniques have been used to optimize the large design space of QCLs. Genetic algorithms (GAs) have been employed to increase the wall-plug efficiency of mid-infrared QCLs [26] and to fine-tune THz QCL transition frequencies across multi-terahertz ranges [27,28,29,30,31]. Simulated annealing has maintained transition energies around 50 meV in triple-step quantum wells [32] and optimized superstructure gratings for nonequidistant frequencies [33]. Inverse spectral theory has been used to maximize gain in quantum well lasers [34] and to optimize the active region of 12 μm QCLs [35]. Machine learning (ML) has been applied to detect defects during fabrication [36,37,38] and to design nanostructures in nanophotonics [39,40]. For QCLs, ML has been used to accelerate modal-gain calculations [41], predict resonant-mode features and emission spectra [42,43], and identify relevant wavefunctions [44]. Recently, an inverse-forward network approach helped predict active region designs and related metrics such as energy differences and LO lifetimes, with errors between 2% and 15% [45], and a ML framework was developed to forecast and optimize QCL designs based on a figure of merit involving threshold gain and lasing transition traits, enabling quick exploration of large design spaces with errors below 15% [46]. Automated wavefunction detection has enabled rapid characterization of key electronic states and performance predictions for interband cascade lasers, greatly reducing the manual analysis of band-structure simulations [47]. For quantum cascade lasers, automated state classification has helped create large, labeled datasets for supervised learning [48], while inverse neural network models have been used to directly map target device metrics to structural parameters, facilitating efficient exploration of complex heterostructure design spaces [49].

Different computational techniques have been successfully applied to the predictive simulation of complex heterostructures such as superlattices and intersubband devices, such as Nonequilibrium Green’s Functions (NEGF), Boltzmann-Bloch, hybrid NEGF and Boltzmann Equation Solvers [50,51,52,53,54,55].

In this work, we use the density matrix (DM) approach [56,57,58,59,60], a computationally efficient quantum transport framework that incorporates coherence effects and accurately describes resonant tunneling across the injection barrier. Due to these capabilities, the DM model is particularly well-suited for the extensive optimization of THz QCL active-region designs.

The DM model is used to calculate transport characteristics, including how material gain, current density, and emission frequency depend on the applied bias. To build the training dataset for ML, we start with the reference high-temperature THz QCL that achieved pulsed operation at 261 K [14]. Variations in each layer’s thickness are introduced, up to ±40% of the nominal value, while the electric field is swept from 17.5 to 27.5 kV/cm. The transport characteristics calculated by the DM for these designs form the dataset used in this study. The trained models are then tested on a separate subset of the dataset to evaluate their ability to predict unseen QCL designs.

To make these predictions, ML techniques are employed, using algorithms capable of identifying patterns in data and generating predictions without explicit manual programming [61]. The primary objective of this work is not merely to compare ML algorithms, but to develop an accurate and computationally efficient surrogate modeling framework capable of approximating density-matrix simulations during large-scale exploration of the QCL configuration space. To achieve this, Random Forest (RF), XGBoost, and Artificial Neural Networks (ANNs) were employed as representative nonlinear predictive models suitable for high-dimensional physical systems. RF and XGBoost were chosen as robust ensemble learning methods capable of capturing nonlinear parameter interactions with good generalization performance, while ANNs were included for their flexibility in approximating highly nonlinear mappings between structural parameters, electric bias, and transport characteristics in QCL heterostructures. The suitability of nonlinear models is further supported by the weak to moderate linear correlations observed between input and output variables, as discussed in Section 3.1.

The trained ANN is employed across the entire configuration space to evaluate how reliably and efficiently it can explore and predict the performance of QCL designs. By screening approximately 44 million configurations, this method enables quick identification of structures with improved material gain and effective filtering of designs based on predicted material gain, current density, and emission frequency.

## 2. Materials and Methods

### 2.1. Starting QCL Design and Data Set

**Figure 1:**
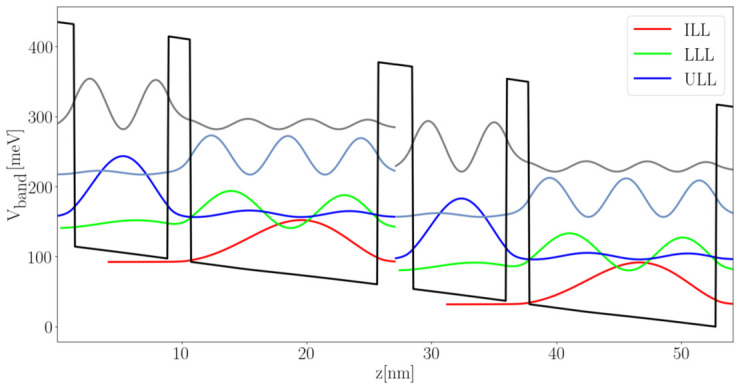
261 K record high-temperature LO-phonon THz QCL [14].

As the initial design for our study, we examine the record-high temperature LO-phonon THz QCL operating at 261 K and emitting at 4 THz, which uses Al_0.35_Ga_0.65_As barriers with an Al molar fraction of x = 0.35 [14]. Efficient depopulation of the lower laser level (LLL) is achieved through longitudinal optical phonon scattering into the injector lower level (ILL), which is 48 meV below the LLL at the resonant bias. The layer thickness sequence, starting from the injection barrier, is **2.88**/7.45/**1.76**/(3.0 + 3.0 + 9.0) nm, with Al_0.35_Ga_0.65_As barriers highlighted in bold, and the middle section of the last quantum well, doped to $1.5\times 10^{17}$ cm^−3^, underlined. Figure 1 shows the conduction-band potential profile of the 261 K record LO-phonon THz QCL, along with the squared wavefunctions.

As QCLs are unipolar devices whose optoelectronic performance depends on the layer thicknesses and the applied bias, the dataset was generated by independently varying each layer thickness up to ±40% of its nominal value, with a step size of 0.7 nm and a thickness variation range from −2.8 nm to +2.1 nm. Simultaneously, the applied electric field is varied from 17.5 to 27.5 kV/cm in 0.5 kV/cm increments.

The statistical analysis was conducted on a dataset of 62,250 samples. Each sample is defined by six layer width parameters, external electric field strength, material gain, frequency, and current density. The first layer width parameter corresponds to the injection barrier, the second to the first well layer, and the third to the second barrier layer. The fourth, fifth, and sixth layer-width parameters all correspond to different sections of the second well layer; however, this layer is divided into three parts because only layer 5 is doped at a concentration of 1.5 × 10^17^ cm^−3^.

The combinations of layer thicknesses and electric fields define a comprehensive set of QCL designs, for which the DM model calculates the material gain, current density, and emission frequency, forming the training data for our machine-learning framework.

### 2.2. Calculating QCL Transport Characteristics Using the Density Matrix Model

Electron transport in THz QCL structures is modeled using the DM formalism [50,51], which enables calculation of key transport quantities, including the material gain g(K), current density J(K), and emission frequency f(K), as functions of the applied electric bias K. The computational method used to determine these quantities is outlined below. For a specific lattice temperature and bias value K corresponding to the device terminal voltage, the Schrödinger–Poisson system is solved self-consistently along with the kinetic balance equations under the equithermal subband approximation, yielding the electronic subband structure and the electron temperature [62]. Using this solution, the DM framework calculates the frequency-dependent gain spectrum g(f) and the current density J. The peak of the gain spectrum g(f) is considered the unsaturated material gain g, while the frequency at this peak defines the emission frequency f. This process is repeated across the entire range of electric bias values K  at a fixed lattice temperature.

From the bias-dependent calculations, the unsaturated material gain is obtained as g(K), while the corresponding current density is determined as J(K). The maximum of g(K) marks the resonant tunneling condition, whereas the peak of J(K) identifies the onset of negative differential resistance (NDR), beyond which lasing typically terminates abruptly. Consequently, QCLs generally operate over the bias range extending from the threshold field, where the material gain equals the threshold value, up to the NDR point, which sets the maximum achievable current density.

**Figure 2:**
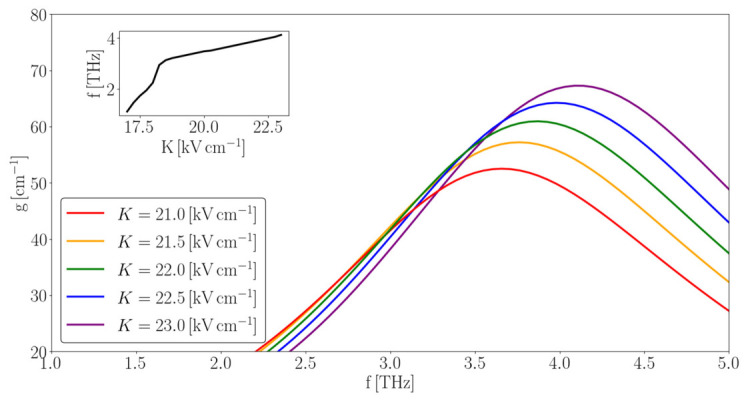
Material gain dependence on emission frequency at different electric biases. The inset of the figure shows the emission frequencies of the peaks of material gain versus the electric bias.

The outlined procedure is illustrated for the record high-temperature LO-phonon THz QCL [14]. Figure 2 shows the frequency-dependent material gain g(f) calculated at several bias values. For each bias, the peak of g(f) corresponds to the lasing mode, and tracking these maxima across bias yields the gain–bias characteristic g(K). The corresponding current density values extracted at each bias define J(K). The inset of Figure 2 displays the dependence of the emission frequency on bias, f(K). The shift in the gain spectra toward higher frequencies with increasing bias arises from the Stark effect.

**Figure 3:**
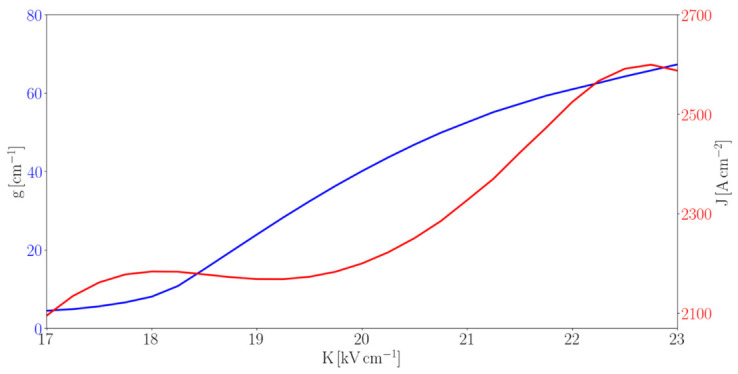
Material gain and current density dependence on electric bias.

Figure 3 summarizes the bias dependence of the material gain g(K) and current density J(K). The calculated material gain increases with electric field and reaches 20 cm^−1^ at approximately 19 kV/cm, becoming comparable to typical waveguide losses and indicating the onset of lasing conditions. The two maxima in the current density originate from two different resonant transport conditions in the QCL structure. The first peak at lower electric fields corresponds to ILL–LLL alignment, where efficient carrier transport occurs without significant population inversion. The second peak at higher fields corresponds to ILL–upper laser level (ULL) alignment, and in this regime population inversion between the ULL and LLL is achieved, producing significant material gain and the regime relevant for QCL operation.

### 2.3. Machine Learning Models

In this study, we use RF [63], XGBoost [64], and ANN [65] to predict material gain, emission frequency, and current density. The models were trained with different layer widths and external electric field strengths as input variables, allowing them to forecast the desired outputs. Although these models are based on different theoretical foundations and computational frameworks, they often play similar predictive roles across various fields, including energy systems modeling, materials science, climate analysis, and industrial optimization.

RF reduces variance through ensemble averaging, and XGBoost enhances predictive performance via gradient-boosted regularization, while ANN architectures use neural representation learning to model complex nonlinear relationships. The most suitable model depends on data characteristics, interpretability needs, available computational resources, and specific application goals. RF is an ensemble method that builds multiple decision trees, each trained on a random subset of data and features, with the overall prediction derived from averaging all trees’ outputs. By combining weak learners into one ensemble, this approach reduces overfitting and boosts the model’s ability to generalize to new data. Conversely, XGBoost employs the gradient boosting framework, sequentially constructing trees that focus on correcting the errors of previous ones. It uses regularization techniques to prevent over-complexity, thereby increasing accuracy and robustness. Additionally, it leverages parallel processing, efficient memory use, and optimized handling of missing data, making it highly scalable for large datasets. ANNs operate under a different paradigm, inspired by biological neurons. They consist of layers of interconnected nodes that transform input data through nonlinear activation functions. In this study, the ANN model used a multilayer perceptron architecture, a type of ANN in which all nodes are fully connected and organized in multiple layers. During training, ANNs update connection weights via backpropagation to minimize prediction errors. They excel at capturing highly nonlinear relationships and learning deep hierarchical representations, making them suitable for tasks like image classification, time-series forecasting, and modeling complex physical phenomena. However, ANNs require large datasets, long training times, and careful hyperparameter tuning to reach optimal performance.

**Figure A1:**
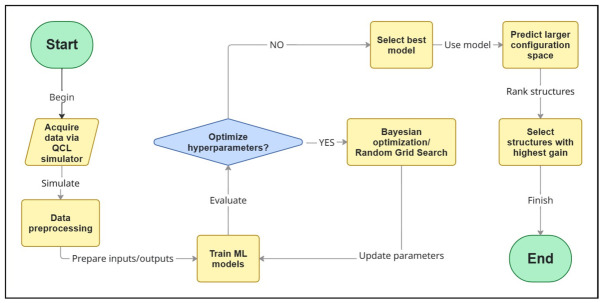
A schematic diagram summarizing the machine learning processes—from data acquisition and preprocessing to model training and prediction.

In this paper, all models were developed in Python 3.11. RF and XGBoost were run within the Jupyter Notebook environment, while the ANN model was run in the built-in Python virtual environment (venv). The overall workflow of the proposed ML framework, from data acquisition and preprocessing to model training and prediction, is summarized in Figure A1 in Appendix A.

## 3. Results and Discussion

### 3.1. Data Analysis, Feature Distributions and Correlations

**Figure 4:**
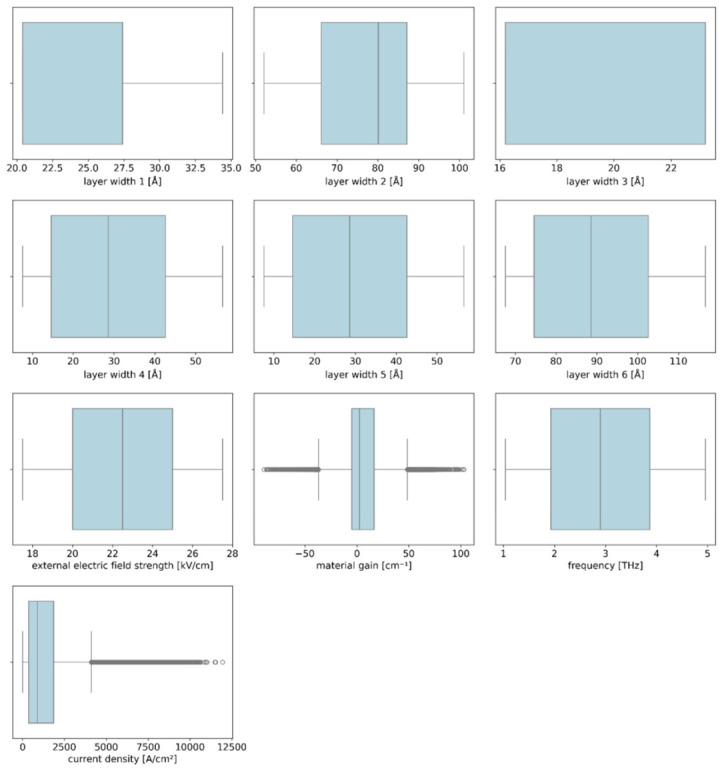
Box plots of layer widths, field strength, material gain, emission frequency, and current density.

The statistical analysis was conducted on a dataset of 62,250 samples, and the distributions of all variables are shown in Figure 4 using box plots.

The six-layer width parameters showed distinct distribution patterns across the samples. The mean layer widths ranged from 19.61 Å for layer 3 to 91.59 Å for layer 6. Layer 2, with a mean thickness of 78.05 Å and a standard deviation of 14.47 Å, demonstrated moderate variability, positioned between the tightly controlled thinner barrier layers. Layers 1 and 3 had relatively low dispersion (standard deviations of 5.63 Å and 3.50 Å, respectively), indicating tighter control over their thicknesses, as expected for barrier layers. In contrast, layers 4, 5, and 6 exhibited substantially higher variability, with standard deviations exceeding 15 Å, suggesting increased structural flexibility or design diversity in these regions. The interquartile ranges further confirmed that layers 4 and 5 have nearly identical thickness distributions, implying coordinated structural tuning.

The external electric field strength had a mean value of 22.43 kV/cm with a narrow spread (standard deviation 3.03 kV/cm). The range extended from 17.5 kV/cm to 27.5 kV/cm, indicating that the applied field was varied within a limited operational range. The box plot confirmed the uniformity of the field distribution. The material gain exhibited a broad, asymmetric distribution, with a mean of 6.48 cm^−1^ and a relatively large standard deviation of 19.93 cm^−1^. Material gain values ranged from −89.31 cm^−1^ to 103.09 cm^−1^, indicating the coexistence of absorptive and amplifying regimes in the dataset. The high variability and the presence of outliers (4.64%) suggest a strong dependence on multiple structural parameters. The interquartile range extended from −4.12 cm^−1^ to 22.51 cm^−1^, indicating that most samples achieved moderate gain. The emission frequency values were concentrated within a relatively narrow range, from 1.04 THz to 4.96 THz, with a mean of 2.91 THz and a standard deviation of 1.13 THz. The approximately symmetric distribution suggests stable spectral operation governed primarily by the quantum well structure and layer composition. The current density exhibited the broadest dynamic range, ranging from 11.75 A/cm^2^ to nearly 11,940 A/cm^2^, with a mean of 1399 A/cm^2^ and a large standard deviation of 1509 A/cm^2^. Outliers accounted for 6.48% of the samples, indicating the presence of extreme cases or nonlinear behavior under specific structural and field conditions. The box plot confirmed a highly skewed distribution, demonstrating that while most configurations produce moderate current densities, a small subset exhibits exceptionally high conduction.

**Figure 5:**
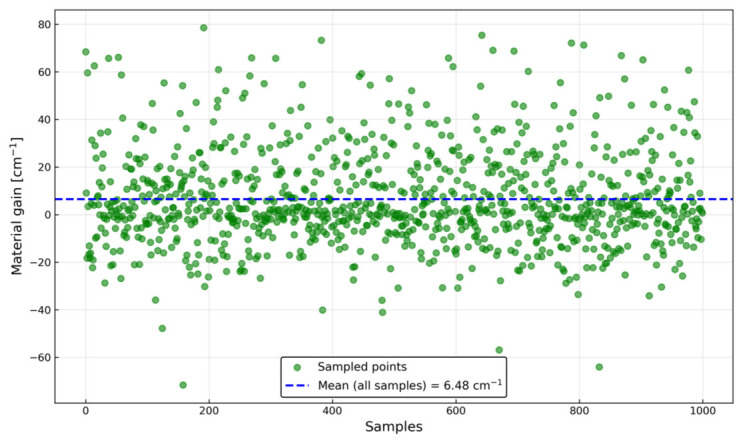
Distribution of material gain with the overall mean indicated.

**Figure 6:**
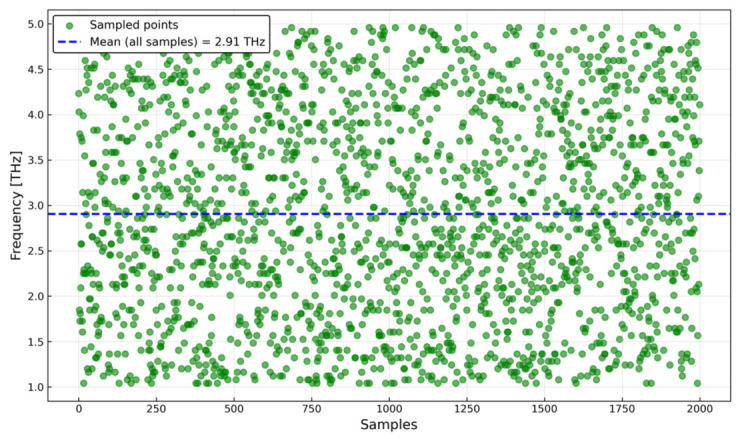
Distribution of emission frequencies with the overall mean indicated.

**Figure 7:**
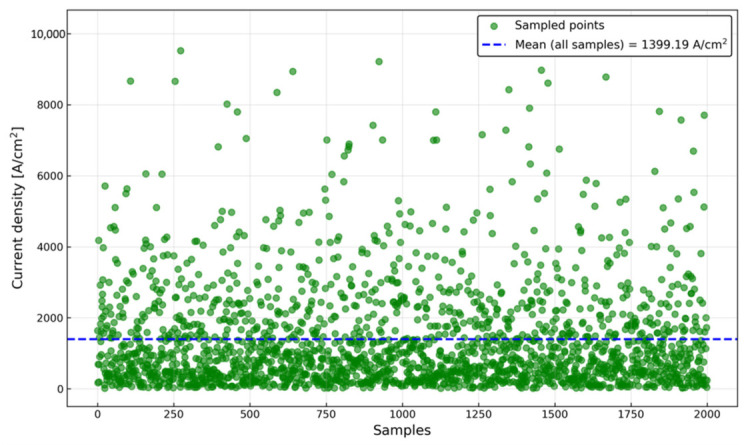
Distribution of current density with the overall mean indicated.

Figure 5, Figure 6 and Figure 7 illustrate the distributions of material gain, emission frequency, and current density for a random subset of 1000 samples, selected for visual clarity and to prevent overcrowding. In each figure, green points represent individual sampled values, emphasizing the variability within the dataset. Figure 5 shows material gain, with the dashed blue line indicating the mean of 6.48 cm^−1^ across the entire dataset, demonstrating that most samples cluster around this overall mean. However, individual points still cover a broad range of values, reflecting the impact of structural variability and other inherent factors.

Figure 6 shows the emission frequency distribution, with the overall dataset mean of 2.91 THz marked by the blue dashed line.

Figure 7 depicts current density, with a mean of 1399 A/cm^2^ across all samples (blue dashed line). The distribution exhibits greater spread and a few extreme values, indicating that while most configurations yield moderate currents, some reach exceptionally high levels.

**Figure 8:**
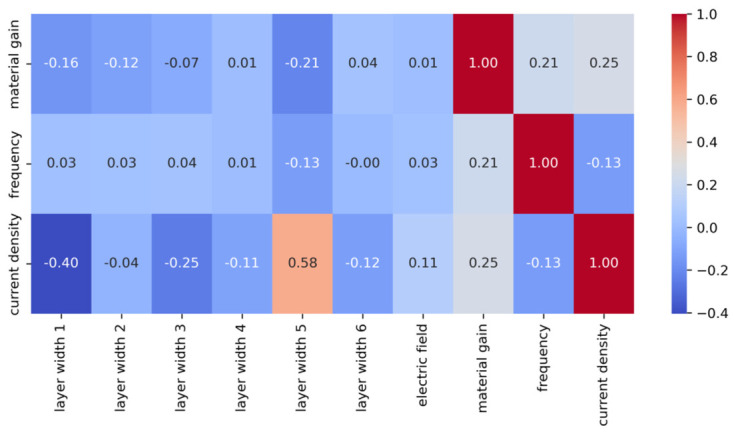
Heatmap showing Pearson correlation between input parameters and target variables.

The Pearson correlation coefficient is commonly used in exploratory data analysis to identify potential dependencies between features and target variables, providing insight into which variables may directly influence each other [66]. Figure 8 presents the Pearson correlation heatmap for all input parameters and target variables. The heatmap highlights the strength and direction of linear relationships, with red denoting positive correlations and blue denoting negative correlations. The correlation matrix for the considered dataset reveals generally weak linear relationships among most features and targets, which can be summarized as follows:

- Material gain shows a weak negative correlation with layer width 5 (r = −0.21), while correlations with other layers are very weak (r ≤ 0.2). The negative correlation between the material gain and the thickness of the doped central region of the second well layer (layer width 5) is explained by increased ionized impurity scattering. Although the doping concentration is kept constant, increasing the thickness of the doped region increases the total sheet doping and extends the spatial overlap between the dopants and the lower laser level wavefunction. This enhances ionized impurity scattering, broadens the intersubband transition linewidth, and increases effective dephasing. Together, these effects reduce the coherent polarization and steady-state gain, explaining the observed negative correlation between material gain and doped-region thickness across the varied structures.
- Frequency is very weakly correlated with all input parameters, and shows the largest correlation with layer width 5 (r = −0.13). Increasing the thickness of the doped region in the second well affects the laser transition frequency by modifying the local potential profile and the spatial distribution of the electron wavefunctions. This changes the energies of the lower and upper laser levels, shifting the intersubband transition energy.
- Current density exhibits moderate correlations with layer widths 1 and 5 (r = −0.40 and 0.58, respectively), a weak correlation with layer width 3 (r = −0.25), and very weak correlations with the other layer widths. The current density increases with the thickness of the doped region in the second well (layer width 5) because a thicker doped layer provides a higher carrier sheet density, thereby enhancing injection into the upper laser level. In contrast, increasing the thickness of the barrier layers (layer widths 1 and 3), and particularly the injection barrier (layer width 1), reduces the current by lowering the tunneling probability between adjacent wells. Thicker barriers reduce wavefunction overlap, thereby limiting carrier transport through the cascade and decreasing the overall current.

While some layer widths have a moderate influence on current density, the absolute values of the Pearson correlation coefficients do not exceed 0.6, with the maximum observed value being 0.58. This indicates that linear models would capture only a small fraction of the observed variability. Overall, these low-to-moderate correlation values suggest that linear dependencies are generally insufficient to fully describe the dataset’s behavior. This clearly justifies the use of nonlinear ML models (RF, XGBoost, and ANN) to predict material gain, emission frequency, and current density. By employing these approaches, predictive accuracy can be significantly improved compared to linear models, which are inherently limited by the low correlations observed.

### 3.2. Machine Learning Models for Predicting Material Gain, Emission Frequency, and Current Density

RF, XGBoost, and ANN models were employed to predict three output parameters of QCL structures: material gain, emission frequency, and current density. The dataset contained 62,250 samples, which were randomly split into a training subset of 43,575 samples (70%), a validation subset of 9337 samples (15%) for hyperparameter tuning, and a test subset of 9337 samples (15%) for final model assessment. Kolmogorov–Smirnov two-sample statistical tests confirmed that the training, validation, and test subsets were drawn from statistically equivalent distributions (*p* > 0.05 for all features), demonstrating the absence of sampling bias during dataset splitting. Hyperparameter tuning was carried out using different optimization strategies according to the learning algorithm. For the Random Forest and XGBoost models, a random search was performed over a predefined hyperparameter space, with 2000 randomly sampled configurations evaluated for each model. The search space included key model parameters such as tree depth, number of estimators, learning rate (for XGBoost), subsampling ratios, and regularization parameters. For the ANN model, Bayesian optimization was used to efficiently explore the continuous hyperparameter space, focusing on both architectural parameters (number of hidden layers and neurons) and optimization parameters (learning rate, batch size, and weight decay). After hyperparameter tuning, the final models were trained with the best parameters for each learning algorithm, and their generalization performance was assessed on both the training and test datasets.

For material gain prediction, the optimal RF model was configured with 250 estimators, a maximum tree depth of 27, a minimum sample split of 2, a minimum of 1 sample per leaf, a feature sampling ratio of 0.9, and bootstrap enabled. This setup yielded Root Mean Square Error (RMSE) values of 2.75 cm^−1^ on the training set and 7.19 cm^−1^ on the test set. The top-performing XGBoost regressor, optimized with a learning rate of 0.08, a maximum depth of 13, 140 estimators, a gamma of 0.3, and a minimum child weight of 5, achieved RMSE values of 1.55 cm^−1^ on the training data and 6.07 cm^−1^ on the test data. The optimal ANN model was trained for 150 epochs and consisted of four hidden layers with 64, 128, 128, and 256 neurons, respectively. The network was trained using the Adam optimizer with a learning rate of 6.59 × 10^−4^, weight decay of 4.40 × 10^−6^, and a batch size of 64. ReLU activation functions were applied in the hidden layers, while a linear activation function was used in the output layer. This model achieved the lowest test RMSE of 1.13 cm^−1^, outperforming the tree-based models. These results are summarized in Table 1.

**Figure 9:**
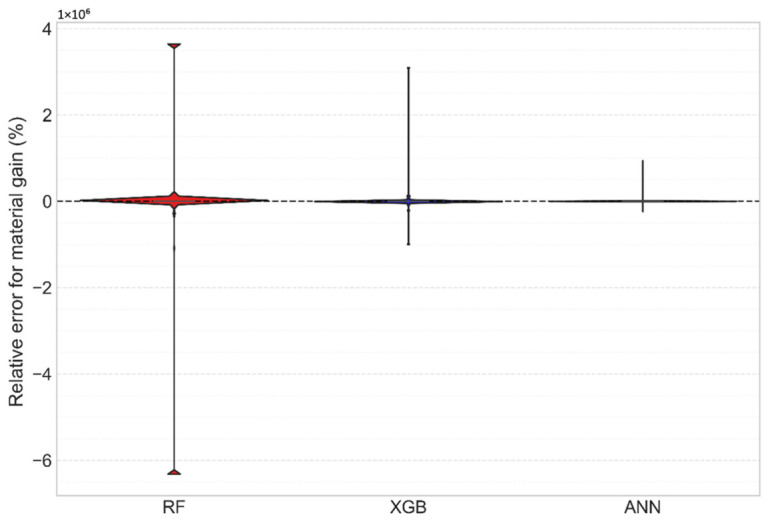
Distribution of relative prediction errors for ML models in material gain prediction.

To further evaluate the predictive performance of the developed models, the distribution of relative prediction errors was analyzed. Figure 9 shows the violin-box plot analysis of the relative prediction errors, highlighting clear differences in the stability and robustness of the evaluated approaches. The ANN model exhibits the narrowest interquartile range with a median relative error of approximately 0.55%, indicating that most predictions were closely aligned with the reference values. In contrast, the RF and XGB models show negative median errors of approximately −5.88% and −3.36%, respectively, demonstrating a systematic tendency towards underprediction. Therefore, the ANN model achieved the most accurate and stable performance, with a median error below 1%, which is considered highly satisfactory for this regression problem.

Additionally, the standardized RMSE relative to the standard deviation of the target variable (SRMSE) was 0.36 for RF, 0.30 for XGBoost, and 0.06 for the ANN model. These results confirm that all models achieve good predictive accuracy (SRMSE < 0.4), with the ANN model demonstrating significantly better performance (SRMSE < 0.2).

For emission frequency prediction, the RF model was configured with 250 estimators, a maximum depth of 27, a minimum sample split of 2, a minimum of 1 sample per leaf, and a feature sampling ratio of 0.9. This setup resulted in RMSE values of 0.23 THz on the training set and 0.62 THz on the test set. The best-performing XGBoost regressor, optimized with a learning rate of 0.1, a maximum depth of 12, 130 estimators, and a minimum child weight of 5, achieved RMSE values of 0.27 THz on the training data and 0.58 THz on the test data. For emission frequency prediction, the best-performing ANN configuration was achieved after 150 training epochs and comprised four hidden layers with 32, 256, 256, and 16 neurons, respectively. The training procedure used the Adam optimizer with a learning rate of 9.58 × 10^−4^, weight decay of 7.622 × 10^−6^, and a batch size of 32. ReLU activation was applied in the hidden layers, while a linear activation function was used in the output layer. The resulting model achieved a test RMSE of 0.25 THz, outperforming all tree-based methods. The corresponding RMSE values are presented in Table 2.

**Figure 10:**
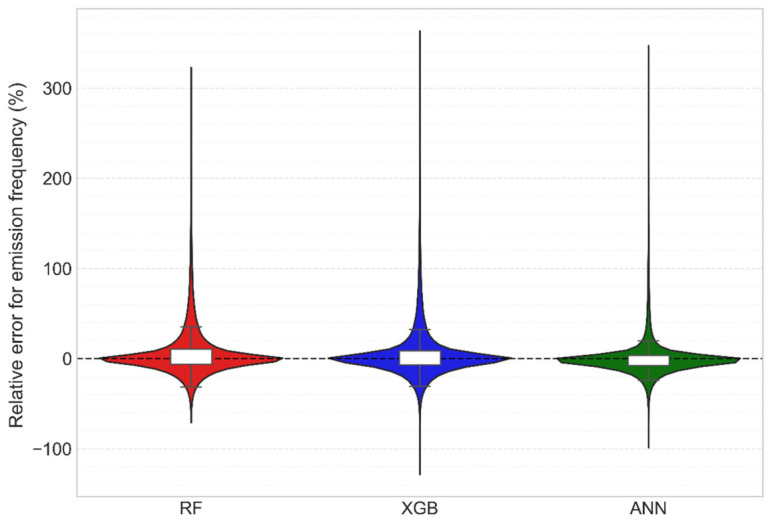
Distribution of relative prediction errors for ML models in emission frequency prediction.

The performance of the models for emission frequency prediction is further examined through the error distribution shown in Figure 10. All three models have median errors close to zero, indicating no strong systematic bias. However, differences are more apparent in terms of dispersion, where the ANN model shows a noticeably tighter interquartile range than the RF and XGBoost models, reflecting greater consistency across samples. Despite this overall stability, all models show extreme deviations in the error tails, with values exceeding 300%, which is attributed to the sensitivity of the relative error metric to small reference frequencies. The ANN model offers the most balanced performance, combining lower RMSE with improved error stability for emission frequency prediction.

The corresponding SRMSE values were 0.55 for RF, 0.51 for XGBoost, and 0.22 for ANN. While all models exhibit acceptable predictive performance, the ANN model clearly outperforms the tree-based approaches. As SRMSE values below 0.40 are generally considered solid indicators of predictive accuracy, these results demonstrate that the ANN model provides reliable and precise emission frequency predictions.

For current density prediction, the RF model employed 250 estimators, a maximum depth of 27, and a feature sampling ratio of 0.9, achieving RMSE values of 118.8 A·cm^−2^ on the training set and 320.8 A·cm^−2^ on the test set. The best-performing XGBoost regressor, tuned with a learning rate of 0.15, a maximum depth of 12, 170 estimators, gamma of 0.2, and a minimum child weight of 4, reached RMSE values of 52.2 A·cm^−2^ for the training data and 247.1 A·cm^−2^ for the test set. The optimal ANN model for current density prediction is composed of four hidden layers with 32, 256, 128, and 32 neurons, respectively. The model was trained using the Adam optimizer with a learning rate of 1.16 × 10^−3^, a weight decay of 1.99 × 10^−4^, and a batch size of 32. ReLU activation functions were applied in the hidden layers, while a linear activation function was used in the output layer. This model achieved the most accurate test predictions, with an RMSE of 55.0 A·cm^−2^, outperforming both tree-based models. These results are summarized in Table 3.

**Figure 11:**
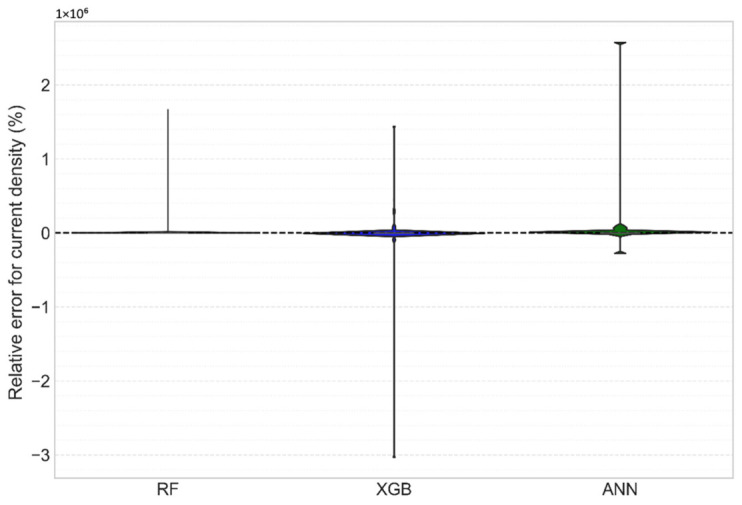
Distribution of relative prediction errors for ML models in current density prediction.

In addition to RMSE-based evaluation, the distribution of relative prediction errors in Figure 11 highlights notable differences in model stability. Although all models have relatively low median errors close to zero, their distributions differ significantly in spread and asymmetry. The ANN model has the most compact interquartile range and greater robustness, while RF and XGBoost show wider dispersion and stronger skewness in the error distribution. The RF model, in particular, exhibits a highly skewed distribution, where extreme outliers compress the main body of the violin plot, making the central distribution appear less pronounced. These results confirm that the ANN model provides the most stable and accurate predictions for current density among the evaluated approaches.

SRMSE values across the current-density range were 0.21 for RF, 0.16 for XGBoost, and 0.04 for ANN, confirming that all models provided accurate predictions, with the ANN model delivering the most precise estimates.

### 3.3. ANN Performance on the Entire Configuration Space

The results in the previous section show that the ANN outperformed both RF and XGB in predicting material gain, current density, and emission frequency on the test subset. In this section, the performance of the ANN is further evaluated across the entire configuration space. Its ability to deliver reliable results is demonstrated by significantly reduced computational time compared to the numerical solver used to generate the training dataset for the ML models.

The mean absolute percentage error (MAPE) between the ANN predictions and the numerical solver results is used as a metric to assess the model’s reliability. For comparison, the MAPE values obtained for the ANN on the test subset for material gain, current density, and emission frequency, presented in the previous section, are summarized in Table 4.

Simulating a single configuration with the numerical solver takes about 20 s. In comparison, the ANN model can generate roughly 250,000 configurations in the same time. Using this method, an algorithm was created to explore the configuration space and identify the 500 best structures that meet a specific criterion.

The construction of the configuration space involved varying each layer’s width by up to ±40% of the nominal value in steps of 2.825 Å (monolayer width), and adjusting the electric field from 17.5 kV/cm to 27.5 kV/cm in 0.5 kV/cm increments. Additionally, because the last three layers (4, 5, and 6) represent the second well layer, only variations resulting in a total width within ±40% of the nominal width of this well layer were considered. The variation parameters for each feature are listed in Table 5.

With this number of combinations for each feature, the total number of configurations in the configuration space was 9 × 22 × 5 × 2106 × 21 = 43,783,740, roughly 44 × 106 configurations. Each configuration was evaluated by each ANN to predict the respective value. The criteria for selecting the best 500 structures were a current density between 1000 A·cm^−2^ and 3000 A·cm^−2^ and maximum material gain.

The entire process of generating all possible combinations, running them through the networks, and saving the best 500 configurations out of approximately 44·106 took 314.4 s. In comparison, simulating the entire dataset (62,250 configurations) took 11.24 h. This provides an approximately 130 times faster way to explore the full configuration space than simulating the complete dataset, and about 90,000 times faster to estimate a single configuration using an ANN than the simulator.

Algorithmically, due to the large number of configurations, it is impossible to keep all of them in memory during the sweep, so a method was developed to process configurations in batches. In this way, only 1,000,000 configurations are processed per batch, resulting in a constant space complexity.

To further validate the accuracy of the ANN across the entire configuration space, the numerical solver was run on the 500 best configurations. The MAPE between the ANN predictions and the corresponding numerical results was calculated for all three output variables and is shown in Table 6.

The parameters of the five configurations with the highest predicted material gain are listed in Table 7, while Table 8 shows the ANN predictions and the corresponding numerical simulator results for material gain, emission frequency, and current density.

It can be observed that in all designs shown in Table 7, both the injection barrier and the second barrier (layers 1 and 3) are generally lowered compared to the initial design, while the widths of the well layers (layers 2 and 4 + 5 + 6) are increased relative to the original configuration. Thinner barrier layers may result in enhanced carrier injection into the upper laser state, which may contribute to increased optical gain. In addition, wider well regions can modify the energy spacing and wavefunction overlap between the relevant subbands, potentially improving the optical transition strength, suggesting that the ANN optimization process captures physically relevant dependencies present in the DM generated dataset.

As shown in Table 8, the predicted values of material gain and emission frequency closely match the results from the numerical simulator. While the predicted current density values show slightly larger deviations, they generally stay within a ±15% relative error range.

For comparison, the values obtained from the numerical simulator for the initial structure corresponding to the design with a record operating temperature of 261 K, which served as the starting point for generating the varied configurations and the entire configuration space, are 64.23 cm^−1^ for material gain, 2591.12 A·cm^−2^ for current density, and 3.685 THz for the emission frequency, all at an electric field of 22.5 kV·cm^−1^.

By training the ANN and applying it to the configuration space, we could reliably identify configurations with higher material gain than the initial design. Additionally, the predicted results for all configurations can now be filtered to optimize, maximize, or select any of the quantities predicted by the ANN, such as material gain, current density, and emission frequency. Since the entire configuration space of approximately 44 million structures has been evaluated using the ANN, this approach allows for an unprecedented speed in searching and analyzing configurations—roughly 90,000 times faster than the simulator—while maintaining a high level of prediction reliability.

## 4. Conclusions

In this work, RF, XGBoost, and ANN were applied to predict the key performance characteristics of THz QCLs, namely material gain, current density, and emission frequency. The models were trained using a dataset generated with a numerical simulator based on the density-matrix transport model, enabling the development of a surrogate modeling framework for rapid evaluation of QCL structures.

The obtained results demonstrated that all investigated ML models achieved reliable predictive performance for material gain, emission frequency, and current density, while the ANN model consistently demonstrated the best overall accuracy. For material gain prediction, the SRMSE values were 0.36 for RF, 0.30 for XGBoost, and 0.06 for ANN. For emission frequency prediction, the corresponding SRMSE values were 0.55 for RF, 0.51 for XGBoost, and 0.22 for ANN, indicating acceptable predictive capability. For current density prediction, the SRMSE values were 0.21 for RF, 0.16 for XGBoost, and 0.04 for ANN. Overall, the ANN model achieved the highest predictive reliability across all investigated performance characteristics.

The trained ANN model was subsequently applied to the full high-dimensional configuration space defined by the considered layer thicknesses and electric fields. Approximately 44 million configurations were evaluated in about five minutes, corresponding to a speedup of roughly 90,000 times compared to the numerical simulator for a single configuration. Analysis of the 500 configurations with the highest predicted material gain resulted in MAPE values of 2.27% for material gain, 4.42% for emission frequency, and 18.9% for current density, confirming that the ANN provides reliable estimates across the explored design space.

The presented approach enables efficient exploration and optimization of QCL configurations while significantly reducing computational cost. In particular, the developed surrogate model allows for rapid identification of structures with improved material gain and provides a practical framework for accelerating the design and optimization of high-temperature THz QCLs.

The proposed framework is limited by the parameter ranges and device structures included in the training dataset, meaning that prediction reliability may decrease for configurations outside the explored domain. In particular, the model has been trained and validated only within the considered quantum-well thicknesses and bias field ranges used in the dataset generation, and its extrapolation to significantly different QCL architectures has not been assessed. Future work may include extending the approach to broader QCL architectures.

## Figures and Tables

**Table 1:** RMSE performance of the models in predicting material gain.

	Training Data [cm^−1^]	Test Data [cm^−1^]
**Random forest**	2.75	7.19
**XGBoost**	1.55	6.07
**ANN**	0.94	1.13

**Table 2:** RMSE values for model predictions of emission frequency.

	Training Data [THz]	Test Data [THz]
**Random forest**	0.23	0.62
**XGBoost**	0.27	0.58
**ANN**	0.19	0.25

**Table 3:** Performance of different models in terms of RMSE for current density.

	Training Data [A⋅cm^−2^]	Test Data [A⋅cm^−2^]
**Random forest**	118.8	320.8
**XGBoost**	52.2	247.1
**ANN**	47.9	55.0

**Table 4:** The mean absolute percentage error between the predicted and actual values from the test dataset for each output characteristic.

Material Gain [%]	Emission Frequency [%]	Current Density [%]
10.55	5.31	4.24

**Table 5:** Minimal value, maximal value, step, and the number of variations generated for every input feature used in the configuration space sweep.

Feature	Minimal Value	Maximal Value	Step	Number of Combinations
Layer width 1	17.28 Å	39.88 Å	2.825 Å	9
Layer width 2	44.7 Å	104.025 Å	2.825 Å	22
Layer width 3	10.56 Å	21.86 Å	2.825 Å	5
Layer width 4	18.0 Å	40.6 Å	2.825 Å	2106
Layer width 5	18.0 Å	40.6 Å	2.825 Å	
Layer width 6	54.0 Å	124.625 Å	2.825 Å	
Electric field	17.5 kV/cm	27.5 kV/cm	0.5 kV/cm	21

**Table 6:** The mean absolute percentage error of predicted values compared to actual values from the 500 best configurations for each output value.

Material Gain [%]	Emission Frequency [%]	Current Density [%]
2.27	4.42	18.9

**Table 7:** Structure parameters of the top 5 configurations with the highest gain, predicted by the ANN, as well as the structure parameters for the initial record high temperature design.

Index	Layer 1 [Å]	Layer 2 [Å]	Layer 3 [Å]	Layer 4 [Å]	Layer 5 [Å]	Layer 6 [Å]	Electric Field [kV/cm]
1	25.755	78.6	13.385	18	20.825	116.15	24
2	25.755	78.6	13.385	20.825	20.825	113.325	24
3	28.58	81.425	13.385	18	18	124.625	21
4	25.755	81.425	13.385	18	18	121.8	22
5	25.755	84.25	13.385	18	20.825	124.625	20
Initial 261 K design	28.8	74.5	17.6	30.0	30.0	90.0	22.5

**Table 8:** Predicted and actual parameters of the top 5 configurations with the highest gain, as predicted by the ANN, along with the parameters for the initial record high temperature design.

Index	Predicted Gain [cm^−1^]	Actual Gain [cm^−1^]	Predicted Frequency [THz]	Actual Frequency [THz]	Predicted Current [A⋅cm^−2^]	Actual Current [A⋅cm^−2^]
1	86.94	89.73	4.22	4.43	2984.65	2426.12
2	86.36	89.22	4.23	4.43	2995.86	2448.40
3	86.16	92.36	3.87	4.11	2526.74	1860.54
4	86.01	88.23	3.78	3.95	2887.73	2301.16
5	85.99	88.72	3.52	3.63	2891.24	2252.85
Initial 261 K design	**Actual Gain [cm^−1^]**		**Actual Frequency [THz]**		**Actual Current [A** **⋅** **cm^−2^]**	
	64.23		3.685		2591.12	

## Data Availability

Data underlying the results presented in this paper are not publicly available at this time but may be obtained from the authors upon reasonable request.

## Abbreviations

The following abbreviations are used in this manuscript:

ANN	Artificial Neural Networks
CW	Continuous Wave
DM	Density Matrix
GAs	Genetic algorithms
ILL	Injection Laser Level
LLL	Lower Laser Level
LO	Longitudinal Optical
MAPE	Mean Absolute Percentage Error
ML	Machine Learning
NDR	Negative Differential Resistance
NEGF	Nonequilibrium Green’s Function
QCL	Quantum Cascade Lasers
RF	Random Forest
RMSE	Root Mean Square Error
SRMSE	Standardized Root Mean Square Error
THz	Terahertz
XGBoost	Extreme Gradient Boosting

## Appendix A

Figure A1 shows the overall workflow used in this study. The process begins with data acquisition and preprocessing, including feature preparation and splitting the dataset into training, validation, and test subsets. Hyperparameter optimization was performed for all machine learning models, and the final models were trained and validated using the resulting optimal configurations. The trained models were then used to predict material gain, emission frequency, and current density, and their performance was evaluated using statistical error metrics. The best-performing machine learning model was further applied to rapidly predict QCL characteristics across a large number of structural configurations, enabling efficient exploration of the configuration space.
